# Body composition and sexual hormones for the glucose control of autoimmune diabetes in males: are they necessary to predict diabetes-related complications?

**DOI:** 10.3389/fendo.2023.1283057

**Published:** 2023-12-21

**Authors:** Mireia García Ramírez, Ángel Rebollo Román, Rafael Palomares Ortega, Rosario Alonso-Echague, María Luisa Calle-Castro, María Ángeles Gálvez Moreno, María José Molina Puerta, Aura D. Herrera-Martínez

**Affiliations:** ^1^ Maimonides Institute for Biomedical Research of Cordoba (IMIBIC), Córdoba, Spain; ^2^ Endocrinology and Nutrition Service, Reina Sofia University Hospital, Córdoba, Spain; ^3^ General Surgery Service, Reina Sofia University Hospital, Córdoba, Spain

**Keywords:** autoimmune diabetes, microvascular complications, glucose control, fat mass, sexual hormones

## Abstract

**Background:**

Glucose control in diabetes is essential for avoiding diabetes-related complications.

**Aim:**

To determine the impact of body composition and sexual hormones in glucose control and diabetes-related complications, in males with autoimmune diabetes.

**Patients and methods:**

Thirty-nine patients with autoimmune diabetes and flash glucose monitoring were included. A morphofunctional nutritional evaluation with bioelectrical impedance vector analysis (BIVA), abdominal adipose tissue ultrasound, rectus femoris ultrasound and biochemical parameters, was performed

**Results:**

Strong, positive correlations were observed between body composition parameters, biochemical variables and sexual hormones (p<0.05). Adipose tissue measured by BIVA and ultrasound was more significantly associated with glucose control (including time in range >70%, glucose variability <36% determined by flash glucose monitoring; p<0.05) and the presence of microvascular/macrovascular complications (p<0.05) than lean mass. After adjusting by the duration of diabetes, BMI, abdominal circumference, fat mass and phase angle increased the risk for microvascular complications (OR 1.32(1.00 – 1.73), OR 1.06(1.00 – 1.12), OR 1.14(1.01 – 1.20), 0R 0.3(0.10 – 0.91) respectively; for macrovascular complications: BMI OR 1.38(1.04 – 1.84) and fat mass OR 1.26(1.00 – 1.58)]. Sexual hormone levels did not influence on glucose control or the development of diabetes-related complications.

**Conclusion:**

Anthrpometric parameters, especially adipose tissue, were associated with glucose control and variability determined by flash glucose monitoring. Furthermore, changes in fat and lean mass were associated with the presence of microvascular and macrovascular complications. Thus, a comprehensive nutritional evaluation might be useful for the evaluation of males with autoimmune diabetes, in order to identify patients with increased risk of complications.

## Introduction

Appropriate glucose control is essential for patients with autoimmune diabetes, in order to avoid microvascular and macrovascular complications. According to a large, recent study in Spanish population, microvascular and macrovascular complications affect 41.14% and 5.83% of patients with type 1 diabetes (T1DM) respectively (1); the same is observed in 23.1% and 4.9% of patients with late autoimmune diabetes of the adulthood (LADA) respectively (2), these complications are associated with decreased quality of life and increased mortality and medical costs.

Both micro and macrovascular complications are more prevalent in males (1, 3). Several factors have been associated with the presence of these complications, specifically, the time of duration of diabetes, glucose control and glucose variability among others (4).

In this context, medical approaches that improve glucose control are always a matter of interest. Current medical options for autoimmune diabetes are based in treatment with insulin; despite the numerous advances in the formulation, duration and stability of several types of insulins (5), the most significant change in the management of diabetes is due to continuous and recent advances in diabetes-applied technologies, including glucose monitoring systems (6, 7). Among them, flash glucose monitoring is the most extended system, probably due to its lower cost, easy-to-use system and trends of glucose levels (7). Specifically, it provides clinical information that is currently recommended for the evaluation of glucose control according to the current clinical guidelines (8), including parameters as: time in range (TIR), time above range (TAR), time below range (TBR), glucose management indicator (GMI) and coefficient of variability (CV). According to recent studies, this system has improved glucose control especially in patients treated with insulin (7, 9), representing a valuable tool for the treatment of autoimmune diabetes.

Additionally, the burden of overweight and obesity currently affects all countries worldwide, and is even observed in diseases that have not been previously related to them. Specifically, T1DM has been classically associated with a non-obese phenotype. Some authors suggest that intensive insulin therapy may be an underlying factor for the increase in body weight and fat (10). Moreover, despite visceral adipose tissue (VAT) has been associated with increased cardiovascular risk, especially in type 2 diabetes (T2DM), it is not routinely evaluated in the clinical practice to predict complications (11).

Furthermore, according to some studies, changes in sexual hormone levels have been associated with metabolic parameters and the presence of microvascular complications (12). In this context, we aimed to perform a study that included a novel morphofunctional nutritional evaluation of patients with T1DM and LADA, to determine its usefulness to evaluate glucose control (using flash glucose monitoring), and/or the presence of diabetes-related complications (microvascular and macrovascular). This evaluation included bioelectrical impedance vector analysis (BIVA), abdominal adipose tissue ultrasound, rectus-femoris (RF) ultrasound, functional tests and biochemical markers, among other parameters, and additional evaluation of male sexual hormones was included.

## Materials and methods

### Patients

This study was approved by the Ethics Committee of the Reina Sofia University Hospital (Cordoba, Spain; reference number CB01072018), which was conducted in accordance with the Declaration of Helsinki and according to national and international guidelines. This is a cross-sectional study, wherein a written informed consent was signed by every individual before inclusion into the study. All patients received information before the inclusion and only if accepted to participate, were included. The inclusion criteria were: males, age > 18 y-old, previously diagnosed with autoimmune diabetes who used flash glucose monitoring (FreeStyle Libre, Abbott Diabetes Care, Witney, United Kingdom). Thirty-nine consecutive patients, evaluated during a twelve-month period, accepted to participate and were included.

### Clinical evaluation

Flash glucose monitoring was used in all patients, glucose control variables of the previous two weeks were collected. This study evaluated: percentage of sensor use, the number of scans per day, mean glucose, GMI, CV, TIR (70-180 mg/dL), TAR (180-250 mg/dl), TAR >250 mg/dL and TBR (54-70 mg/dl); time below 54 mg/dL was 0% in most patients and consequently was not included in the analysis.

Microvascular and macrovascular complications are routinely evaluated in our outpatient clinic according to the current clinical guidelines (8). Specifically, all patients undergo to an annual digital screening of diabetic retinopathy (13); for the diagnosis of diabetic kidney disease, the ration albumin/creatinine is determined at least once per year, if positive (albumin/creatinine ratio >30 mg/g), 2 of 3 spot urine samples within 3 to 6 months are elevated to confirm the diagnosis (14). For the diagnosis of peripheral neuropathy, regular screening is performed with sensory testing and confirmed with peroneal conduction velocity and sural nerve action potential amplitude. Cerebrovascular complications were considered only if the patient presented with a brain computed tomography or magnetic resonance imaging (MRI) with ischemic lesions; cardiovascular complications were considered only if the patient presented with coronary artery lesions in MRI, angiography or diagnostic/therapeutic coronary artery catheterization; peripheral artery disease was considered only in patients with artery obstruction in doppler ultrasound.

Nutritional evaluation was performed by the same endocrinologist in all cases. Height was determined using a calibrated wall-mounted height rod, weight was determined using a bioelectrical impedance (TANITA MC-780MA). Body mass index (BMI) was computed as body weight (Kg)/height (m^2^). Mid-arm circumference was measured at the midpoint between the tip of the acromion and the olecranon process on the nondominant side of the body using a flexible tape measure; calf circumference was measured at the midpoint of a 90° folded leg. Additionally, vectorial bioelectrical bioimpedance (BIVA) was performed using a NUTRILAB-Akern impedanciometer, for handgrip strength, a Jamar® hydraulic dynamometer was used and the nutritional ultrasound was performed using a GE Logiq E9 ultrasound machine and a linear 9L-D probe. Adipose tissue (AT) of the abdomen and RF-ultrasound were performed as previously described (15, 16). Abdominal ultrasound evaluated: global adipose tissue (GAT), subcutaneous AT, superficial subcutaneous AT, deep subcutaneous AT and VAT (A representative schema is depicted in **Supplementary Figure 1**). RF-ultrasound evaluated: adipose tissue, RF-cross sectional area, RF-circumference, RF-Y axis and RF-X axis. BIVA-measured variables were: body cell mass (BCME), extracellular mass (ECME), fat mass, lean mass, water, bone mass and phase angle. Functionality was evaluated using the timed up and go test (TUG).

For evaluating androgen deficiency-related symptoms, the Adm questionary was used (17). Specifically, patients self-reported their affective state using a subjective scale (good, not so good and bad); patients were asked about their capacity to perform their regular activities (yes/no question), decreased muscle strength (yes/no question) and decreased libido (yes/no question).

Biochemical parameters included hemoglobin, lymphocytes, albumin, prealbumin, ferritin, transferrin, 25-OH vitamin D, total cholesterol, LDL-cholesterol, HDL-cholesterol, C-reactive protein (C-RP) and triglycerides. Regarding hormone levels, thyrotropin (THS), follicle-stimulating hormone (FSH), luteinizing hormone, androstenedione, testosterone and dehydroepiandrosterone sulphate (DHEAS.)

### Statistical analysis

Between-group comparisons were analyzed by the Mann–Whitney U test (nonparametric data), or the Kruskal–Wallis test (nonparametric data, when we compared more than two groups). Paired analysis was performed by Student t Wilcoxon test (nonparametric data). Chi-squared test was used to compare categorical data. Additionally, logistic regression analyses were included in this study for obtaining the odds ratio (OR) and 95% confidence intervals (CIs). Specifically, a logistic regression analysis adjusted by the duration of diabetes was initially performed for microvascular complications, macrovascular complications and GMI<7 since this variable was statistically significant in the univariate analysis, additionally, it was adjusted by duration of diabetes, tobacco exposure, hypertension and dyslipidemia since this variables have been previously associated with microvascular and macrovascular complications. Statistical analyses were performed using SPSS statistical software version 20, and Graph Pad Prism version 6. Data are expressed as median, interquartile range and percentages. p-values <0.05 were considered statistically significant.

## Results

### Autoimmune diabetes, comorbidities and diabetes-related complications

Thirty-nine male patients were included, with a median age of 53 years old. Most patients had T1DM (74.4%). LADA patients were significantly older than T1DM patients (median age 57 vs 46 years old respectively), and the duration of diabetes tended to be shorter in them (p=0.07). The presence of comorbidities, macrovascular, microvascular and other clinical characteristics were comparable in both groups. Specifical characteristics of the included patients are depicted in **Table 1**.

**Table 1:** General characteristics of the included patients.

Characteristic	All patients (n=39)	T1DM (n=29)	LADA (n=10)	*p*
Age	53 (40 - 61)	46 (39 – 60)	57 (54 – 68)	0.02
Time since diagnosis (years)	21 (11.5 - 29)	24 (13 – 33)	11.5 (8.7 – 5.5)	0.07
Tobacco exposure				0.17
Non smoker	81.6 (31/39)	75.8 (22/29)	90 (9/10)	
Active smoker	10.5 (4/39)	10.3 (3/29)	10 (1/10)	
Former smoker	7.9 (3/39)	10.3 (3/29)	0	
Complications				
Hypertension	28.2 (11/39)	31.8 (7/22)	40(4/10)	0.33
Dyslipidemia	33.3 (13/39)	48.3 (14/29)	60 (6/10)	0.52
Microvascular complications	33.3 (13/39)	60 (12/29)	10 (1/10)	0.06
Macrovascular complications	15.4 (6/39)	17.2 (5/29)	10 (1/10)	0.58
Weight loss (previous 6 months)	33.3 (13/39)	37.9 (11/29)	20 (2/10)	0.30
Total weight loss (kg)	4 (2 - 4.75)	5.5 (2.2 – 9.2)	1.5 (1.2 – 1.7)	0.18
Gastrointestinal symptoms	5.1 (2/39)	5.1 (2/29)	0	0.39
Self-reported affective state				0.36
Good	84.6 (33/39)	66.7 (26/29)	70 (7/10)	
Not so good	12.9 (5/39)	6.8 (2/29)	30 (3/29)	
Bad	2.6 (1/39)	3.4 (1/29)	0	
Clinical variables				
Decreased muscle strength	17.9 (7/39)	17.2 (5/29)	20 (2/10)	0.84
Routine physical activity	50 (19/39)	48.3 (14/29)	50 (5/10)	0.90
Decreased libido	23.1 (9/39)	24.1 (7/29)	20 (2/29)	0.78

T1DM, type 1 diabetes; LADA, Latent autoimmune diabetes in adults.

### Type of autoimmune diabetes and glycemic control using flash glucose monitoring

Glucose monitoring (determined by active sensor and number of scans per day) was similar in patients with T1DM and LADA. Additionally, glycemic control determined by GMI, TIR, TAR, TBR was also comparable in both groups (**Table 2**).

**Table 2:** Glycemic control according to flash glucose monitoring.

Characteristic	All patients (n=39)	T1DM (n=29)	LADA (n=10)	*p*
Sensor use (%)	91 (91 – 99)	97 (91 – 99)	97 (95 – 97)	0.51
Self-scanning (number/day)	11 (8 – 17)	12 (9 – 17)	11 (8 – 23)	0.26
Mean glucose (mg/dl)	154 (136 – 174)	155 (137 – 172)	147 (135 – 174)	0.76
GMI (%)	6.95 (6.6. – 7.48)	7 (6.6 – 7.4)	6.8 (6.5 – 7.6)	0.71
CV (%)	33.4 (28.1 – 38.7)	34 (27 – 38)	32 (28 – 38)	0.75
TIR	64 (52 – 76.5)	64 (53 – 72)	62 (51 – 84)	0.84
TAR	22 (14 – 27)	23 (17 – 27)	21 (12 – 25)	0.94
TAR (>250 mg/dL)	5 (1.5 – 12.5)	5 (2 -11)	7.5 (1 – 14)	0.80
TBR	3 (1 – 5)	3 (1 – 5)	3.5 (1 – 5.7)	0.67

GMI, glucose monitoring index; CV, coefficient of variation; TIR, time in range; TAR, time above target range; TBR, time below range.

### Type of autoimmune diabetes, sexual hormones and nutritional composition

Subcutaneous AT and VAT determined by abdominal adipose tissue ultrasound were increased in LADA patients compared with T1DM patients (p<0.05; **Table 3**).

**Table 3:** Morphofunctional nutritional evaluation.

Characteristic	All patients (n=39)	T1DM (n=29)	LADA (n=10)	*p*
Current body weight (kg)	76.6 (71.2 – 90.2)	75.7 (69.9 – 91.3)	77 (72.4 – 86.9)	0.88
Prevalence of overweight				0.22
Normal weight (%)	35.9	68.0	32.0	
Overweight (%)	64.1	85.7	14.3	
BIVA				
BMI	26 (23.9 – 29.3)	25.2 (23.7 – 28.8)	26.8 (26-1 – 29.1)	0.74
BCME	40 (37.5 – 42.6)	39.6 (38 – 42)	40.4 (36.2 – 42.1)	0.78
ECME	21.1 (21.1 – 23)	21.1 (19.9 – 22.8)	21.4 (20.3 – 23)	0.95
Fat mass (%)	20.3 (16.8 – 27.3)	18.8 (16.4 – 27.7)	21.4 (19.5 – 22.8)	0.85
Fat mass (Kg)	15.2 (12.1 – 23.2)	14 (12 – 23)	16.3 (14.8 – 22.6)	0.93
Lean mass (%)	75.7 (69 – 79)	77.1 (69.7 – 79.3)	73.2 (69.3 – 76.5)	0.25
Lean mass (Kg)	75. (69 – 79)	77.1 (69.7 – 79.3)	56.3 (51.3 – 61.8)	0.18
Water (%)	56.1 (52.6 – 59)	56.7 (52.6 – 61)	55.6 (52.8 – 57.3)	0.36
Water (Kg)	43.7 (41 – 47.1)	43.1 (41.1 – 47.1)	42.8 (38.6 – 46.3)	0.17
Bone mass	3.1 (2.9 – 3.3)	3 (2.9 – 3.2)	3.1 (2.8 – 3.2)	0.95
Phase angle	5.8 (5 – 6.6)	6 (5.2 – 6.5)	5.7 (4.7 – 6.6)	0.99
Anthropometric measurements				
Abdominal circumference	99 (91.5 – 106)	97 (90 – 105)	102 (99.2 – 108.5)	0.22
Arm circumference	32 (30 – 33.5)	32 (30 – 34)	31.5 (30 – 32.7)	0.54
Calf circumference	37.5 (35.25 – 40)	38 (36 – 40)	31 (34 – 41)	0.61
Functionality				
Mean handgrip strength	42.6 (36.6 – 49.8)	44 (37 – 50)	38 (36 – 43)	0.20
TUG	8.63 (7.7 – 10.3)	8.9 (7.7 – 10.2)	7.7 (7.3 – 8.6)	0.18
Nutritional echography				
*Rectus femoris echography*				
Adipose tissue (cm)	0.69 (0.54 – 0.96)	0.7 (0.5 – 1.1)	0.6 (0.5 – 0.7)	0.19
RF-cross sectional area (cm2)	4.07 (3.66 – 5.26)	4 (3.7 – 5.5)	4.1 (3.6 – 4.6)	0.53
RF-circumference (cm)	9.56 (8.89 – 10.37)	10 (8.8 – 10.6)	9.3 (8.9 - 9.4)	0.41
RF-Y axis (cm)	1.31 (1.18 – 1.52)	1.3 (1.1 - 1.5)	1.3 (1.2 – 1.5)	0.99
RF-X axis (cm)	4.21 (3.81 – 4.53)	4.3 (3.8 – 4.2)	4 (3.8 – 4.5)	0.55
*Abdominal adipose tissue echography*				
Global adipose tissue (cm)	2.35 (1.75 – 3.489	2.1 (1.6 – 3.1)	3 (2.1 – 3.5)	0.19
Subcutaneous adipose tissue (cm)	1.47 (1.14 – 2.05)	1.4 (1 - 1.9)	1.6 (1.2 – 2.5)	0.05
Superficial subcutaneous adipose tissue (cm)	0.64 (0.46.- 0.93)	0.6 (0.4 - 0.8)	0.6 (0.5 – 0.9)	0.65
Deep subcutaneous adipose tissue (cm)	0.82 (0.61 – 1.07)	0.8 (0.6 – 1)	0.7 (0.6 – 1)	0.86
Visceral adipose tissue (cm)	0.77 (0.46 – 1.080)	0.5 (0.4 – 1)	1 (0.7 – 1.6)	0.01
Biochemical nutritional parameters				
Hemoglobin	15 (14.5 – 15.45)	15 (14.5 – 15.4)	15 (14 – 15.6)	0,84
Lymphocytes	2060 (1659 – 2484)	2070 (1720 – 2520)	1825 (1600 – 2420)	0.55
Albumin (g/dl)	4.8 (4.6 – 5)	4.9 (4.6 – 5)	4.8 (4.6 – 4.8)	0.12
Prealbumin (mg/dl)	22.2 (19 – 24)	21.1 (19 – 24.7)	23.7 (22 – 26.5)	0.15
Ferritin (mg/dl)	81 (42 – 109)	88.3 (47 – 122)	44 (32 – 67)	0.13
Transferrin (mg/dl)	218 (206 – 251)	216 (206 – 234)	266 (219 – 289)	0.01
Total Cholesterol (mg/dl)	181 (153 – 203)	179n (149 – 204)	188 (159 – 190)	0.90
HDL-Cholesterol (mg/dl)	54 (47 – 63)	54 (48 – 61)	57 (46 – 63)	0.61
LDL-Cholesterol (mg/dl)	94 (69 – 122)	97 (7171 – 125)	88 (69 – 112)	0.51
Triglycerides (mg/dl)	128 8 (91 – 176)	128 (84 – 176)	125 (107 – 196)	0.11
C-reactive protein (g/dl)	0.74 (0.5 – 2.9)	1.1 (0.5 – 3)	0.6 (0.5 – 1.1)	0.25
25-OH vitamin D (ng/ml)	22 (17 – 25)	23.2 (18.4 – 25.1)	19.6 (12.7 – 22.5)	0.24
Serum hormone levels				
TSH	1.7 (1.36 – 2.65)	1.7 (1.4 – 2.7)	1.4 (1 – 2.1)	0.08
FSH	5.8 (4.03 – 8.25)	4.5 (3.7 – 7.9)	7.6 (5.6 – 8.4)	0.93
LH	5 (3.6 – 6.4)	5. (3.3 – 6.4)	4.3 (3.7 – 6.4)	0.49
Testosterone	508.5 (392 – 670)	494 (421 – 675)	554 (375 – 642)	0.76
Androstenedione	2.56 (1.93 – 3.19)	2.5 (2.2 – 3.1)	2.9 (1.7 – 3.8)	0.47
DHEAS	144 (87.75 – 204.75)	157.5 (100 – 213)	95.5 (54 – 138)	0.03

BIVA, Bioelectrical impedance vector analysis; BCME, body cell mass; ECME, extracellular mass; TUG, tested up and go; RF, rectus femoris; TSH, thyrotropin hormone; FSH, follicle stimulating hormone; LH, luteinizing hormone; DHEAS, Dehydroepiandrosterone sulphate.

Anthropometric measurements, body composition using BIVA, functional variables and RF-ultrasound were similar in both group of patients. Regarding biochemical variables, transferrin was lower, and DHEAS levels were higher in patients with T1DM (p<0.05), we did not observe differences between other parameters including sexual hormones were similar in patients with LADA and T1DM (**Table 3**).

### Morphofunctional nutritional evaluation in patients with autoimmune diabetes

**Figure 1:**
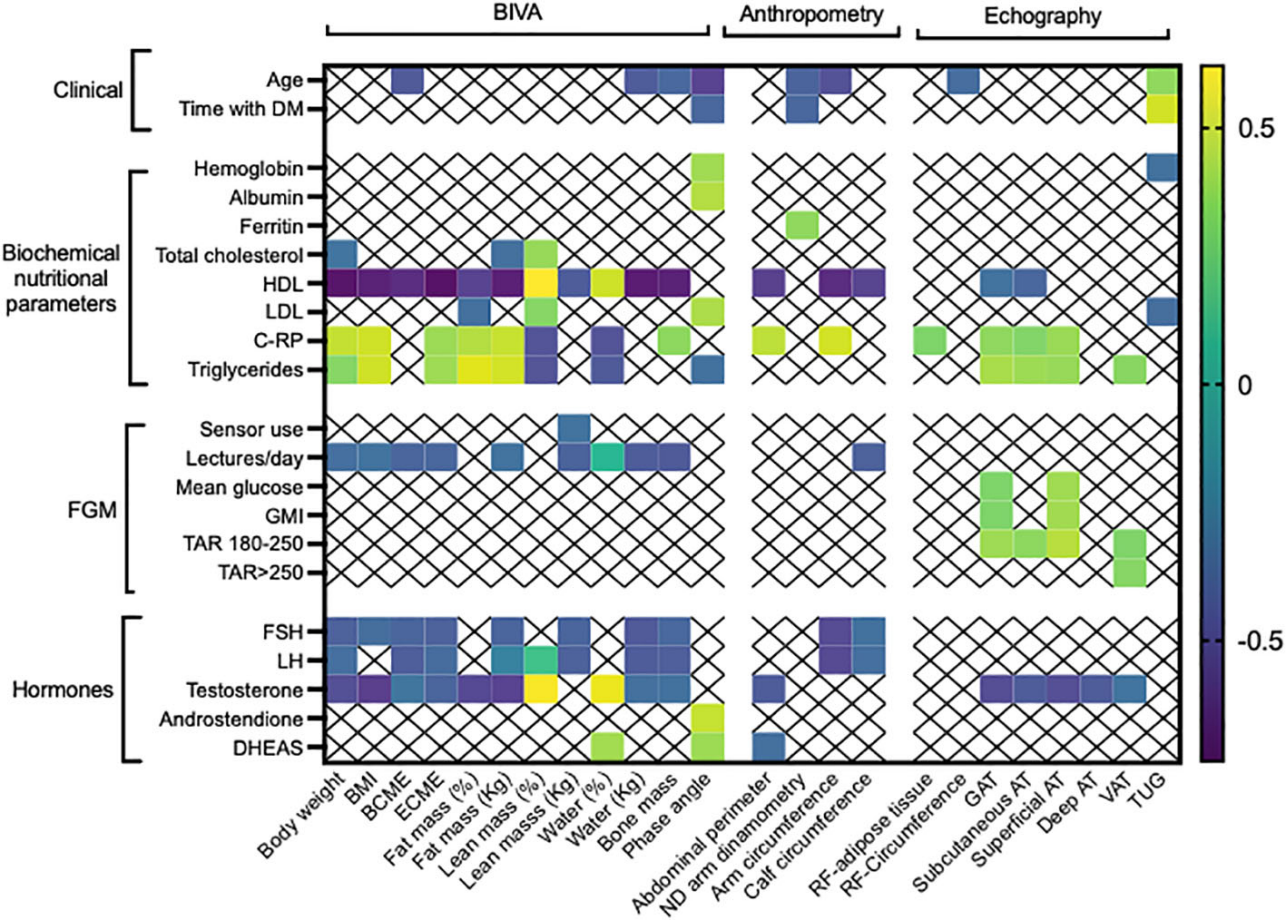
Significant correlations between nutritional parameters (morphofunctional evaluation), glycemic control using flash glucose monitoring and serum sexual hormones. DM, diabetes; BIVA, Bioelectrical impedance vector analysis; BMI, body mass index; BCME, body cell mass; ECME, extracellular mass; ND, non-dominant; RF, rectus femoris; GAT; global adipose tissue; AT, adipose tissue; VAT, visceral adipose tissue; C-RP, C reactive protein; TSH, thyrotropin hormone; FSH, follicle stimulating hormone; LH, luteinizing hormone; DHEAS, Dehydroepiandrosterone sulphate; GMI, glucose monitoring index; TIR, time in range; TAR, time above target range; TBR time below range. Only statistically significant correlations are depicted.

Age was negatively correlated with some body composition parameters using BIVA (BCME, water, phase angle), non-dominant handgrip strength, arm circumference and RF-circumference (**Figure 1**). Similarly, duration of diabetes was negatively correlated with phase angle and non-dominant handgrip strength (p<0.05; **Figure 1**).

Concerning biochemical nutritional parameters, hemoglobin and serum albumin levels positively correlated with phase angle. HDL cholesterol negatively correlated with body weight, BMI, fat mass, arm-, calf-, abdominal circumferences, subcutaneous AT and GAT, it also positively correlated with percentage of lean mass; in contrast, LDL-cholesterol positively correlated with lean mass and phase angle. C-RP positively correlated with body weight, BMI, ECME, fat mass, abdominal perimeter, arm circumference, RF-adipose tissue, GAT, subcutaneous AT, superficial AT and negatively correlated with lean mass and water. Similarly, serum triglycerides correlated in a positive manner with body weight, BMI, ECME, fat mass, phase angle, subcutaneous AT, GAT and VAT (p<0.05; **Figure 1**).

Regarding flash glucose monitoring, patients with a higher number of scans per day presented lower body weight, BMI, BCME, ECME, fat mass and calf circumference. Mean glucose and GMI positively correlated with GAT and superficial AT, while TAR positively correlated with GAT, subcutaneous, superficial AT and VAT, in contrast, TAR > 250 mg/dL positively correlated only with VAT (p<0.05; **Figure 1**).

When sexual hormones were analyzed, FSH and LH negatively correlated with body weight, BCME, ECME, water, fat/lean/bone mass in kilograms, arm and calf circumferences. Serum testosterone levels presented the same correlations and additionally, negatively correlated with subcutaneous AT, superficial AT, deep AT and VAT, furthermore, it positively correlated with percentage of water and lean mass (p<0.05; **Figure 1**). Finally, DHEAS positively correlated with phase angle, water in percentage and negatively with abdominal circumference (p<0.05).

### Glucose control and nutritional composition in autoimmune diabetes

**Figure 2:**
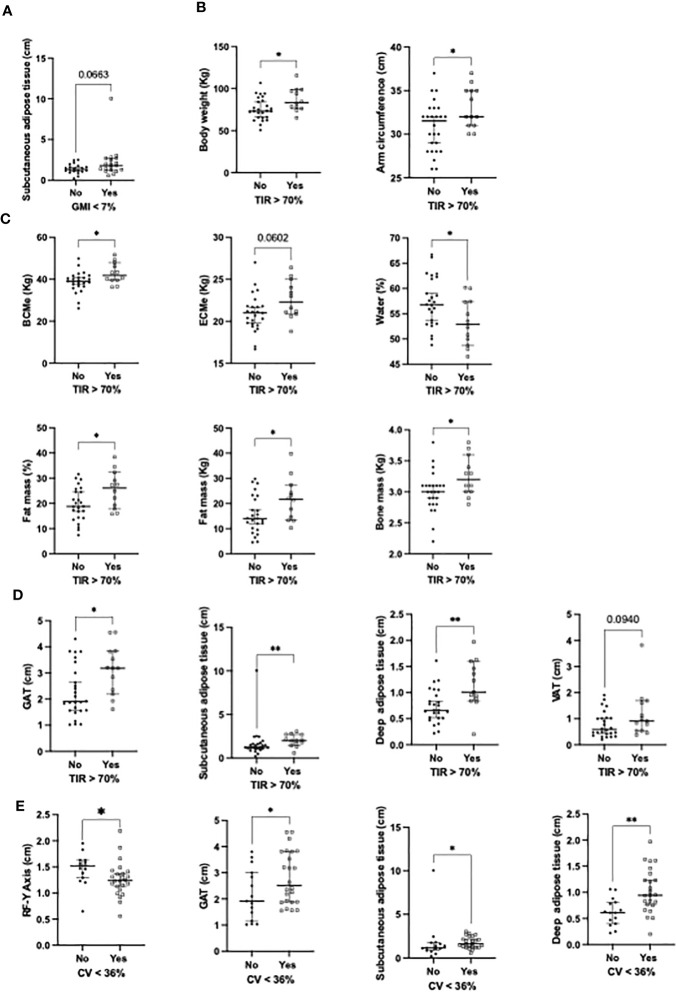
Significant associations between **(A)** nutritional parameters and GMI<7%; **(B)** TIR >70% and clinical variables; **(C)** TIR >70% and bioimpedance variables; **(D)** TIR >70% and ultrasound variables; **(E)** CV<36% and ultrasound variables. RF, rectus femoris; GAT, global adipose tissue; VAT, visceral adipose tissue. *p<0.05; **p<0.01.

An optimal glucose control using flash glucose monitoring was evaluated according to the parameters: GMI<7%; TIR>70% and CV<36%. Patients with GMI<7% tended to present with increased subcutaneous AT (**Figure 2A**). Patients with TIR>70% presented higher body weight, arm circumference, BCME, fat and bone mass (**Figures 2B, C**), additionally, presented with increased GAT, subcutaneous AT, deep AT and VAT compared with patients with TIR<70% (**Figure 2D**). Furthermore, patients with CV<36% presented with decreased RF-Y axis and increased GAT, subcutaneous, and deep AT (**Figure 2E**). A multivariate analysis adjusted by the duration of diabetes revealed that superficial AT was strongly associated with GMI <7% (OR: 16.41, 95% CI: 1.46-183; **Table 4**). This association disappeared when the multivariate analysis was also adjusted by hypertension, dyslipidemia and tobacco exposure. Furthermore, testosterone levels were significantly associated with GMI <7% (OR 1.01, 95% CI 1.02 – 1.03; **Table 5**).

**Table 4:** Multivariate logistic regression of nutritional assessment methods, diabetes-related complications and glucose control adjusted by duration of diabetes.

	Variable	OR	CI	*p*
Microvascular complications	BMI	1.32	1.00 – 1.73	0.02
	Abdominal circumference	1.06	1.00 – 1.12	0.03
	Fat mass (Kg)	1.14	1.01 – 1.29	0.03
	Phase angle	0.3	0.10 – 0.91	0.03
	TUG	3.34	1-28 – 8.66	0.01
Macrovascular complications	BMI	1.38	1.04 – 1.84	0.02
	Body weight	1.10	1.00 – 1.21	0.03
	Fat mass (Kg)	1.26	1.00 – 1.58	0.04
GMI < 7%	Superficial AT	16.41	1.46 -183	0.02

**Table 5:** Multivariate logistic regression of nutritional assessment methods, diabetes-related complications and glucose control adjusted by duration of diabetes, hypertension, dyslipidemia and tobacco exposure.

	Variable	OR	CI	*p*
Microvascular complications	BMI	1.27	0.82 – 1.96	0.29
	Abdominal circumference	1.04	0.95 – 1.14	0.44
	Fat mass (Kg)	1.16	0.89 – 1.53	0.26
	Phase angle	0.02	0.10 – 2.40	0.40
	TUG	3.70	0.29 – 5.29	0.19
	Testosterone	0.99	0.99 - 1.01	0.36
	Androstenedione	1.58	0.55 – 4.49	0.39
	DHEAS	1.01	0.99 – 1.02	0.41
Macrovascular complications	BMI	1.35	0.73 – 2.49	0.33
	Body weight	1.14	0.95 – 1.38	0.16
	Fat mass (Kg)	1.04	0.32 – 3.42	0.95
	Testosterone	1.00	0.99 – 1.01	0.79
	Androstenedione	1.72	0.54 – 5.50	0.36
	DHEAS	1.00	0.98 – 1.02	0.99
GMI < 7%	Superficial AT	0.01	0.003 – 0.42	0.02
	Testosterone	1.01	1.02 – 1.03	0.006
	Androstenedione	1.25	0.66 – 2.37	0.50
	DHEAS	1.01	0.98 – 1.02	0.16

### Microvascular complications, sexual hormones and nutritional composition in patients with autoimmune diabetes

**Figure 3:**
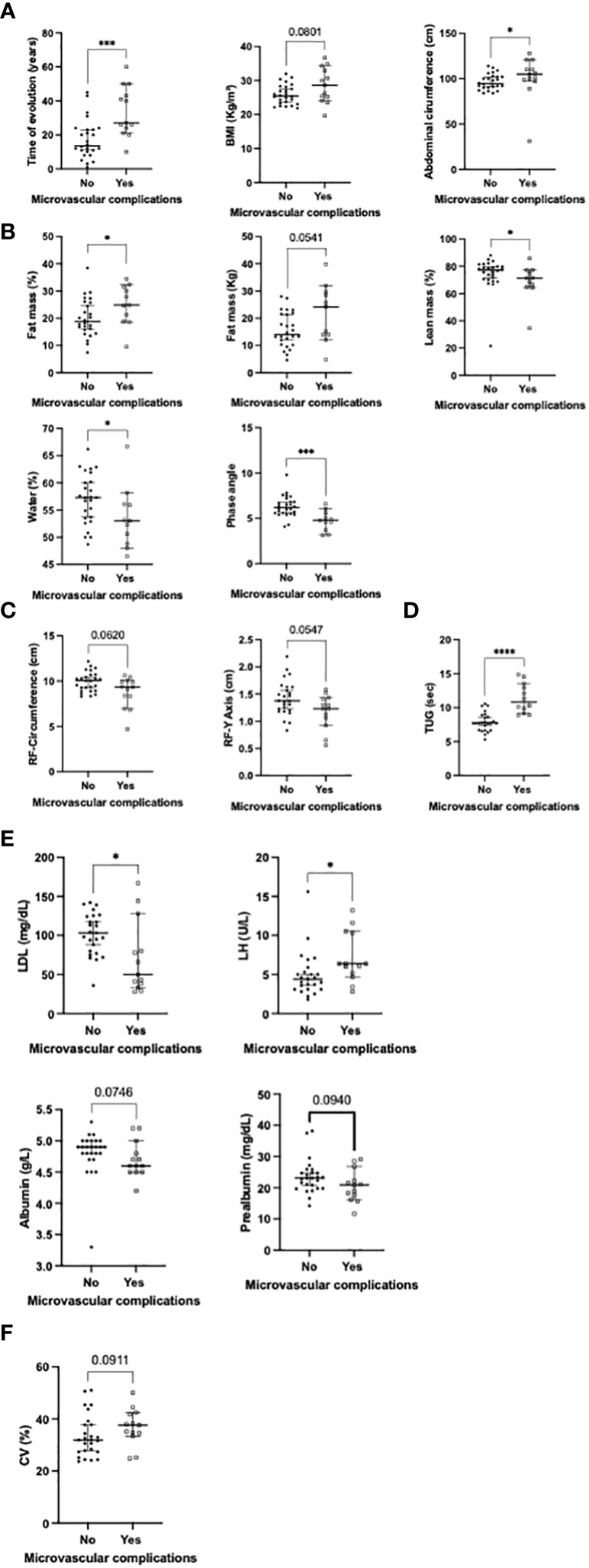
Significant associations between the presence of microvascular complications and: **(A)** clinical variables; **(B)** body composition using BIVA; **(C)** body composition using ultrasound; **(D)** functional tests; **(E)** biochemical parameters; **(F)** Flash glucose monitoring (univariate analysis). BMI, body mass index; TUG, tested up and go; RF, rectus femoris; LH, luteinizing hormone. *p<0.05; ***p<0.001; ****p<0.000.1.

Microvascular complications were associated with increased time of diabetes evolution (median of 27 vs 13.5 years respectively; **Figure 3A**), increased percentage of fat mass in percentage, decreased percentage of lean mass, water and phase angle (p<0.05);. Additionally, abdominal circumference was increased and functionality (determined by the TUG test) was decreased in these patients (p<0.05; **Figure 3D**). Other variables including increased BMI and kilograms of fat mass; decreased RF-circumference and Y axis, tended to be associated with microvascular complications (**Figures 3A-C**). LDL-cholesterol levels were decreased in these patients, additionally, and serum LH levels were increased (p<0.05; **Figure 3E**). Serum albumin and prealbumin tended to be decreased (**Figure 3E**), while CV was increased in patients with microvascular complications (**Figure 3F**). A multivariate analysis adjusted by the duration of diabetes revealed that BMI, abdominal circumference, fat mass (Kg), phase angle and TUG still remained associated with microvascular complications [OR: 1.32 (CI: 1.001 – 1.73), 1.06 (CI: 1.002 – 1.12), 1.14 (CI: 1.01 – 1.29), 0.3 (CI: 0.10 – 0.91) and 3.34 (CI: 1.28 – 8.66) respectively; **Table 4**]. When the multivariate analysis was adjusted by the duration of diabetes, tobacco exposure, hypertension and dyslipidemia this association disappeared, additionally, sexual hormones were not associated with microvascular complications (**Table 5**).

### Macrovascular complications, sexual hormones and nutritional composition in patients with autoimmune diabetes

**Figure 4:**
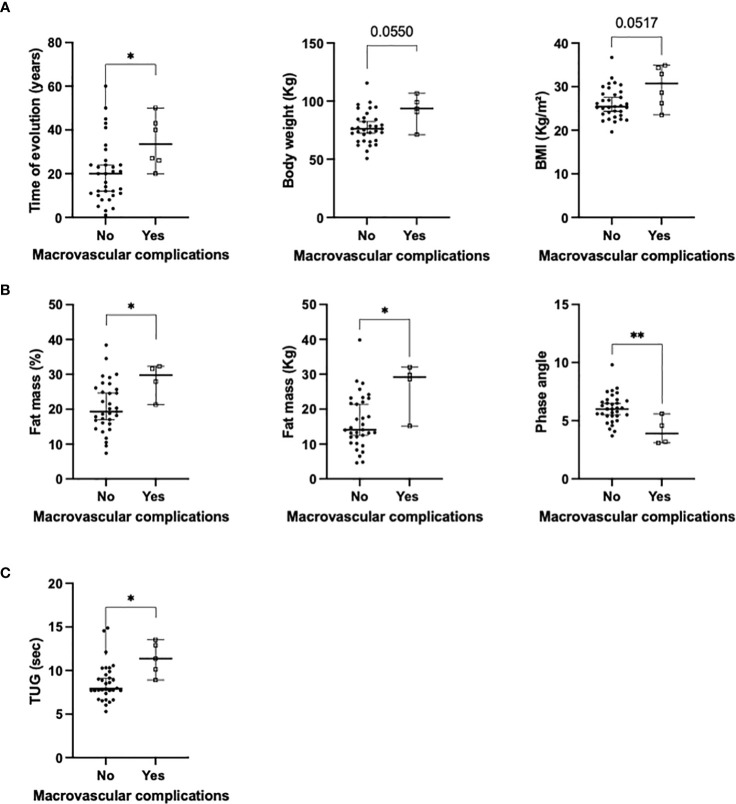
Significant associations between the presence of macrovascular complications and: **(A)** clinical variables; **(B)** body composition using BIVA; **(C)** functional tests (univariate analysis). BMI, body mass index; *p<0.05; **p<0.01.

Macrovascular complications were significantly associated with increased duration of diabetes (median of 20 vs 33 years respectively; **Figure 4A**), increased fat mass (in percentage and absolute kilograms; **Figure 4B**), decreased phase angle and functionality (p<0.05; **Figure 4C**). Increased body weight and BMI tended to be associated with the presence of macrovascular complications. A multivariate analysis adjusted by the duration of diabetes revealed that BMI, body weight and fat mass (Kg) still remained associated with macrovascular complications [OR: 1.38 (CI: 1.04 – 1.84), 1.10 (CI: 1.002 – 1.21) and 1.26 (CI: 1.002 – 1.58) respectively; **Table 4**]. When the multivariate analysis was additionally adjusted by hypertension, tobacco exposure and dyslipidemia, these associations were not statistically significant. Sexual hormones were not associated with macrovascular complications in this analysis (**Table 5**).

## Discussion

Treatment goals in patients with autoimmune diabetes has significantly evolved during the last years, especially due to important advances in diabetes-applied technologies. Current glucose control goals are focused in the prevention of micro and macrovascular complications. Despite this, the general phenotype of the society has changed, with significant increase in overweight and obesity, which can influence the evolution and complications of diseases, including autoimmune diabetes. In this context, this study reports a comprehensive nutritional evaluation of males with autoimmune diabetes, it also included hormone-related variables that should be taken into account for adjusting treatment and predicting glucose control and diabetes-related complications.

Classically, LADA has been compared with type 2 diabetes (T2DM), since most patients are initially considered affected with T2DM. Despite this, current evidence, including genome analysis, suggests that the clinical evolution and control of LADA patients are more comparable to T1DM than to T2DM (18, 19). Based on this, patients with T1DM and LADA were included in this study and complications were adjusted by the duration of diabetes.

Similar to previous studies, most evaluated patients were overweight (20). This is a remarkable fact, since T1DM has been classically associated to a slim phenotype. In this sense, Belgium, and several countries in Europe report a similar prevalence of overweight/obesity in patients with T1DM and the general population (20); in Austria, despite overweight and obesity prevalence is similar in patients with T1DM and the overall population, in 30–49 year-old patients, BMI is significantly higher in those with T1DM (21). In other scenarios, a national T1DM registry in Mexico reported 34.3% overweight and 8.1% obesity (22), in contrast, the prevalence of overweight and obesity is lower in people with T1DM compared with the general population in the USA (23). Regarding LADA patients, previous studies have reported that insulin resistance, overweight and obesity were present, even in comparable proportions with patients with T2DM (24); specifically, in these patients, a younger age at diabetes onset was associated with a slimer phenotype (25). In our cohort, we did not observe patients with obesity, but, 64% presented with overweight, especially patients with T1DM, compared with patients with LADA.

Our study was performed in adults, but this tendency of overweight and obesity has been also described in childhood. A recent meta-analysis described that body fat was 9% higher in children and adolescents with T1DM compared to their peers; specifically, their total body fat mass was increased 1.2 kg, which equivalates to 2.3% (26). These authors suggested that larger daily insulin dosage (U/kg/day) was associated with a larger difference in body fat percentage, while other factors including sex, age, height, body mass index (BMI), HbA1c, or disease duration were not related (26).

In our cohort, BMI and fat mass were clearly related with the presence of micro and macrovascular complications in autoimmune diabetes in the multivariate analysis adjusted by duration of diabetes. In this line, former studies have reported an association between higher percentage of fat mass and metabolic comorbidities in T1DM, including elevated blood pressure and dyslipidemia (27, 28). Furthermore, increased fat mass has been associated with slower muscle re-oxygenation after physical activity in T1DM, and in consequence with insulin resistance and higher cardiovascular disease risk (28).

Previous studies have reported that during the first year of diabetes onset, patients with T1DM increase their fat mass in about 13.3% (1.6 kg) and their lean mass in 4.9% (2.5 kg), which in total, is lower that their perceived weight loss before diagnosis (29). We did not evaluate body weight at diagnosis, but the presence of increased fat mass using BIVA is remarkable.

Similar prevalence of micro and macrovascular complications was observed in patients with T1DM and LADA, as previously reported (2, 19). It has been already described that in both, LADA and T1DM, poorer glycemic control is associated with increased risk of microvascular complications (19). Clinical studies reporting diabetes-related complications in LADA are poor. Specific studies that compare complications in patients with LADA and T1DM are lacking, some comparisons are focused between LADA and T2DM patients. In this sense, 11% of patients included in the United Kingdom Prospective Diabetes Study (UKPDS) presented with autoimmune diabetes; a *post-hoc* analysis reported that these patients had a 27% lower risk of major adverse cardiovascular events compared to patients with T2DM, which was explained by the fact that patients with LADA were younger and had a more favorable cardiometabolic profile (30). In contrast, LADA participants had a 55% lower adjusted risk for microvascular complications during the first nine years after diagnosis, but this lower risk later disappeared due to worse glucose control, specifically, the adjusted risk was 33% greater than in T2DM beyond the first 9 years of follow- up (30).

The novel morphofunctional nutritional evaluation included in this study (31) has been reported in patients with sarcopenia and malnutrition (15, 16), but this is the first report of its application in autoimmune diabetes. Its use provides additional information about body composition and clinical evolution. The evaluation of fat and lean mass is gaining a significant place in the regular evaluation of patients in different scenarios, since sarcopenia (lean mass loss) and fat mass (especially visceral fat) have been associated with increased inflammation, worse clinical outcomes and mortality in patient suffering from different diseases (32–36). Specifically, adipose tissue evaluated by BIVA and ultrasound has been associated with lower glucose variability and increased TIR, probably related with increased insulin resistance as previously reported in patients with T2DM (32, 37). Furthermore, as in patients with obesity (32), adipose tissue and inflammation were correlated, despite C-RP were not above the reference range in the included patients. Importantly, in this study, VAT correlated with triglycerides and TAR, suggesting a higher cardiometabolic risk in T1DM, as suggested in other scenarios including T2DM (38, 39).

Regarding sexual hormones, it is well-known that males with T2DM have lower testosterone levels than the general population (40), even decreased testosterone levels have been associated with insulin resistance (41). Some authors suggest that this association is not present in patients with T1DM (42). Similarly, in our cohort we did not observe testosterone deficiency. Other studies have reported that testosterone levels are mainly associated with BMI (43); not only we did observe this correlation, but also correlations with BMI BCME, ECME and fat mass. Additionally, we did not observe associations between sexual hormone levels or the presence of diabetes-related complications, similarly to a previous report of Condorelli and cols (44). In contrast, we observed an association between testosterone levels and the presence of GMI<7%, in line with a recent study that described a negative correlation between HbA1c and triglycerides levels with total testosterone levels (12).

Regarding specifically diabetes-related complications, it has been suggested an association between elevated testosterone levels and increased risk of diabetic nephropathy during puberty (45), especially if accompanied by poor glycemic control (46). We did not observe relations between microvascular complications and testosterone (or other sexual hormones) levels. Differences might be explained by the fact that our study only included adults.

This study has some limitations, especially the number of participants; we did not include children or adolescents, and obesity was absent in the studied population. In contrast, this study has several strengths: first of all, it is a real reflect of the clinical practice, in which patients with autoimmune diabetes present with disease-related microvascular complications, sexual-related symptoms and also macrovascular complications. All patients used flash glucose monitoring, which provided updated information of glucose control when the nutritional evaluation was performed. Additionally, we performed a comprehensive nutritional evaluation, including anthropometric, ultrasound, functional and biochemical parameters; finally, sexual hormones were also included in order to avoid confounding results.

This study reflects several interesting points in the clinical evolution of patients with autoimmune diabetes. First of all, the time of evolution of diabetes influences not only the presence of microvascular and macrovascular complications, but also affects muscle mass and functionality (reflected with phase angle ad handgrip strength). Additionally, the role of inflammation in type 1 diabetes is obtaining and emerging role, which has some differences and similarities with type 2 diabetes (47), specifically in this study, a chronic inflammatory state was reflected when C-RP levels correlate with adipose tissue-related parameters. Additionally, patients with increased fat mass presented lower testosterone levels, despite hypogonadism was not present, testosterone levels clearly affect several scenarios of life in males, including muscle strength, courage and libido (48). As previously reported, patients with increased body weight and fat mass present an insulin-resistance component (49), which offers patients with T1DM more stability in their glucose control (higher TIR), but it was not been associated with decreased presence of micro/macrovascular complications. Furthermore, patients with microvascular complications presented with worse nutritional status reflected in biochemical parameters (albumin, prealbumin), body composition (increased fat mass and decreased phase angle) and specially decreased functionality (evaluated with the TUG). Finally, flash glucose monitoring was more frequently used in patients with lower body weight and fat mass, which could have been associated with a better self-reported state or the presence of more interest in their health.

The applicability of this novel nutritional evaluation should be evaluated in different scenarios, since it may provide additional information about the clinical status of the patients, their risk of complications and the effect of their quality of life. Despite this, the absence of cutoff points for the ultrasound and the increased time required for physical evaluation could limit their regular use in the clinical practice. In this context, this study reflects the importance, at least, of fat mass and functionality evaluation in patients with autoimmune diabetes, since it could help to early diagnose and treat patients at risk of developing microvascular/macrovascular complications.

## Conclusions

Taken together, our results reveal that the measurement of some anthropometric parameters (especially fat mass using BIA and functionality using TUG) provides additional and valuable information for the evaluation of male patients with autoimmune diabetes. Additionally, adipose tissue measured by ultrasound and bioimpedance analysis was strongly correlated with glucose control and variability. Furthermore, BIVA analysis and functional tests may help identify patients with increased risk of complications. In contrast, the evaluation of sexual hormones did not provide relevant clinical information about glucose control and outcomes in males with T1DM or LADA. Additional studies in larger populations should be performed in order to improve current tools to identify and prevent the development of microvascular and macrovascular complications in patients with autoimmune diabetes.

## Data availability statement

The original contributions presented in the study are included in the article/**Supplementary Material**. Further inquiries can be directed to the corresponding author.

## Ethics statement

The studies involving humans were approved by Reina Sofía University Hospital. The studies were conducted in accordance with the local legislation and institutional requirements. The participants provided their written informed consent to participate in this study.

## Author contributions

AH: Conceptualization, Data curation, Formal analysis, Funding acquisition, Investigation, Supervision, Writing – original draft, Writing – review & editing. MR: Investigation, Writing – review & editing. ÁR: Investigation, Writing – review & editing. RO: Investigation, Writing – review & editing. RA: Investigation, Writing – review & editing. MC: Investigation, Writing – review & editing. MM: Writing – review & editing. MP: Investigation, Writing – review & editing.

## Supplementary material

Supplementary Figure 1 Schematic view of the adipose tissue evaluation in the abdominal nutritional ultrasound
