# Supplementary figures and images for: Body composition and sexual hormones for the glucose control of autoimmune diabetes in males: are they necessary to predict diabetes-related complications?
Source: Front Endocrinol (Lausanne). 2023 Dec 21;14:1283057. doi: 10.3389/fendo.2023.1283057 (PMC10773863; doi:10.3389/fendo.2023.1283057)

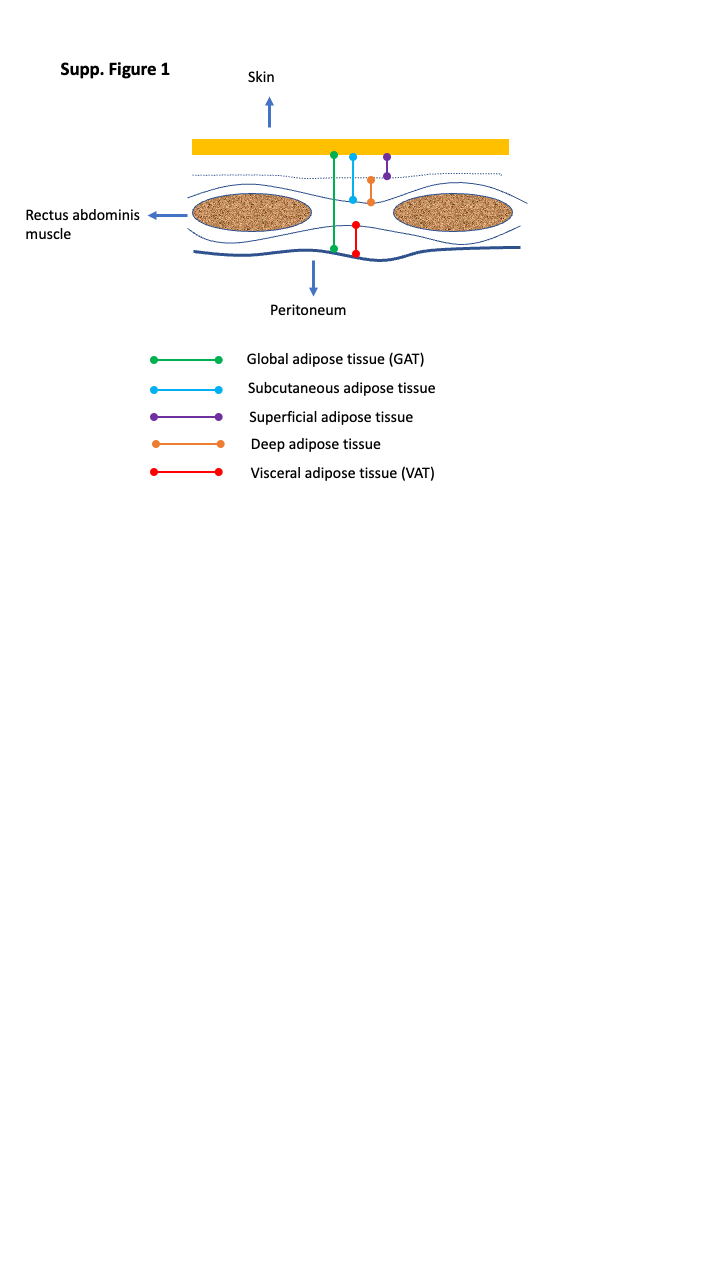

Supplement: Supplementary Figure 1 — Schematic view of the adipose tissue evaluation in the abdominal nutritional ultrasound [file Image_1.tiff]
